# Immersive 3D Virtual Reality Cancellation Task for Visual Neglect Assessment: A Pilot Study

**DOI:** 10.3389/fnhum.2020.00180

**Published:** 2020-05-25

**Authors:** Samuel E. J. Knobel, Brigitte C. Kaufmann, Stephan M. Gerber, Dario Cazzoli, René M. Müri, Thomas Nyffeler, Tobias Nef

**Affiliations:** ^1^Gerontechnology and Rehabilitation Group, University of Bern, Bern, Switzerland; ^2^Perception and Eye Movement Laboratory, Departments of Neurology and BioMedical Research, Inselspital, Bern University Hospital, University of Bern, Bern, Switzerland; ^3^Neurocenter, Luzerner Kantonsspital, Lucerne, Switzerland; ^4^ARTORG Center for Biomedical Engineering Research, University of Bern, Bern, Switzerland

**Keywords:** immersive virtual reality, visual neglect, stroke, cancellation task, head-mounted display

## Abstract

**Background**: Unilateral spatial neglectis an attention disorder frequently occurring after a right-hemispheric stroke. Neglect results in a reduction in qualityof life and performance in activities of daily living. With current technical improvements in virtual reality (VR) technology, trainingwith stereoscopic head-mounted displays (HMD) has become a promising new approach for the assessment and the rehabilitation of neglect. The focus of this pilot study was to develop and evaluate a simple visual search task in VR for HMD. The VR system was tested regarding feasibility, acceptance, and potential adverse effects in healthy controls and right-hemispheric stroke patients with and without neglect.

**Methods**: The VR system consisted of two main components, a head-mounted display to present the virtual environment, and a hand-held controller for the interaction with the latter. The task followed the rationale of diagnostic paper-pencil cancellation tasks; i.e., the participants were asked to search targets among distractors. However, instead of a two-dimensional setup, the targets and distractors were arranged in three dimensions, in a sphere around the subject inside its field of view. Usability and acceptance of the task, as well as the performance in the latter, were tested in 15 right-hemispheric subacute stroke patients (10 of whom with and five of whom without unilateral spatial neglect; mean age: 67.1 ± 10.5 years) and 35 age-matched healthy controls.

**Results**: System usability and acceptance were rated as high both in stroke patients and healthy controls, close to the maximum score of the questionnaire scale. No relevant adverse effects occurred. There was a high correlation (*r* = 0.854, *p* = 0.002) between the Center of Cancellation [an objective neglect measure) calculated from a paper-pencil cancellation task (Sensitive Neglect Test (SNT)] and the newly developed VR cancellation task.

**Conclusion**: Overall, the developed visual search task in the tested VR system is feasible, well-accepted, enjoyable, and does not evoke any significant negative effects, both for healthy controls and for stroke patients. Findings for task performance show that the ability of the VR cancellation to detect neglect in stroke patients is similar to paper-pencil cancellation tasks.

## Introduction

Unilateral spatial neglect is a syndrome that frequently occurs after a stroke. Patients fail to respond to or report stimuli presented in the contralesional space (Heilman and Valenstein, 1981). These impairments are not explainable through deficiencies within the primary sensory or motor systems but arise from an attentional deficit (Mark et al., 1988). Different studies show variable incidence rates of neglect. In the acute state, incidences up to 70% for right-hemispheric and up to 60% for left-hemispheric strokes were reported (Stone et al., 1993; Bowen et al., 1999). The severity of the symptoms often decreases within the first few months after stroke. However, in 25–35% of stroke patients, and especially in right-hemispheric stroke patients, this remission is incomplete (Robertson and Halligan, 1999; Ringman et al., 2004; Corbetta, 2014). In these patients, neglect impairs performance in the activities of daily living, such as getting dressed (e.g., patients may forget to tie the contralesional shoe), grooming (e.g., they may shave asymmetrically or brush their hair only on the ipsilesional side), reading (e.g., missing the beginning of a new text line or reading only the ipsilesional part of words), navigating in space (e.g., collisions with contralesional objects such as doors and poles), and social interaction (e.g., neglecting people appearing within the contralesional space; Buxbaum et al., 2004). Additionally, neglect is an independent, negative prognostic factor for the overall outcome of stroke rehabilitation (Jehkonen et al., 2006).

For the diagnosis of neglect, several assessment tools have been developed. The most commonly used tools are cancellation tasks (Gauthier et al., 1989; Halligan et al., 1989; Reinhart et al., 2016), line bisection (Albert, 1973), and reading tasks (Caplan, 1987). These paper-pencil tasks are widely used because they are easily applicable, do not require much time and effort for the patients, are overall well supported by normative data, and—in the acute to subacute state—they correlate with the patients’ impairments in everyday life, e.g., measured with the Catherine Bergego Scale (Azouvi et al., 1996; Menon and Korner-Bitensky, 2004; Ringman et al., 2004). A commonly used measure to quantify neglect severity in cancellation tasks is the Center of Cancellation (CoC) proposed by Rorden and Karnath (2010). The CoC can be calculated from most cancellation tasks and reflects the normalized mean deviation from the center due to neglect. CoC values range from −1 to 1, whereby zero indicates an unbiased spatial distribution; positive CoC values indicate a shift towards the right, and negative CoC values indicate a shift towards the left side of space. This means a CoC close to 1 (resp. −1) reflects a more severe neglect compared to a CoC of 0.5 (resp.−0.5). One problem of some paper-pencil cancellation tasks, though, is a reduced sensitivity for minor neglect symptoms and a low retest-reliability due to learned compensation (Menon and Korner-Bitensky, 2004).

With the advances in technology, specifically in virtual reality (VR), computer-based assessment methods for the neglect have been increasingly developed. Testing and training using stereoscopic head-mounted displays (HMDs) have been introduced as a promising new and complementary approach for the assessment and the rehabilitation of unilateral spatial neglect (Kim et al., 2011, 2015; Yasuda et al., 2017). Correspondingly, several researchers have proposed to complement the paper-pencil tests with VR tasks (Fordell et al., 2011; Coyle et al., 2015; Pedroli et al., 2015).

As an example of a VR-based neglect assessment, Buxbaum et al. (2012) proposed a new way of detecting neglect, thereby showing evidence that mild neglect is often not detected by common paper-pencil cancellation tasks (i.e., Line-Bisection and Bells Test for the near space, and Laser Line-Bisection for the far space), but can be detected using dynamic 3D, non-immersive VR tasks. The task proposed by the authors was the VR Lateralized Attention Test (Dawson et al., 2008), in which patients were placed in front of a large screen and were navigating along a virtually designed path. The goal of the task was to name the objects appearing on the right and the left side of the virtual path. The main finding was that neglect was detected with higher sensitivity with this VR task compared to the paper-pencil cancellation task. Another approach proposed by Gupta et al. (2000) was presented through a two-case study. In the task, patients wore an HMD, while a scene with different objects was presented to them. The patients were asked to name and count the objects and, congruently with neglect symptoms, failed to report objects on the contralesional side. Dvorkin et al. (2012) investigated how the arrangement of the targets influences the detection rate. They found a higher sensitivity for the VR-based assessment method for polar target arrays compared to linear arrays and paper-pencil tasks. Besides these examples, other studies also showed a higher sensitivity of computerized neglect assessment (Tanaka et al., 2005; Broeren et al., 2007; Aravind et al., 2015). Detailed information regarding these studies is summarized in the review by Ogourtsova et al. (2017).

Overall, VR tasks have several advantages: (1) With its surrounding three-dimensional environment, a VR-based setup can simulate everyday situations and present more complex tasks than it is possible on article. Thereby, it allows providing a safe environment, in which to practice activities of daily living and skills that might be too dangerous to train in reality (e.g., street crossing training, Kim et al., 2007, 2010). (2) The additional dimension available in VR allows examining the extra-personal space, too. Indeed, neglect can affect both peripersonal (within reaching distance of the subject) and extra-personal (outside of reaching distance) space, but these two spatial planes can dissociate (Robertson and Halligan, 1999; Aimola et al., 2012). An additional neglect testing employing VR may be particularly useful in the chronic stage (i.e., more than 6 months after stroke) when paper-pencil methods become less sensitive (Rengachary et al., 2009). Last but not least, by designing gamified tasks and visually appealing environments, neglect assessment, and training using VR can result in an enjoyable experience for the patients. This can increase motivation, allowing for a higher dose and intensity of therapy (Mainetti et al., 2013; Shapi’i et al., 2015; Faria et al., 2016; Tobler-Ammann et al., 2017).

Although the acceptance of state-of-the-art HMDs is generally high, even in elderly participants (Jäger et al., 2015; Cook and Winkler, 2016) and in critically ill patients (Gerber et al., 2017), the occurrence of cybersickness can reduce their usability (LaViola, 2000; Riener and Harders, 2012). The cybersickness syndrome can result from a mismatch between the visual input and the vestibular and proprioceptive input. This mismatch occurs when the user is stationary (e.g., sitting on a chair), while motion is implied by the virtual environment (e.g., the avatar representing the user is moving within the virtual environment). Symptoms of cybersickness are similar to those of motion sickness and include headache, sweating, nausea, eye strain, and dizziness. Cybersickness can be avoided by programming a stationary avatar within the virtual environment (e.g., the avatar and the user both sit in a chair). This minimizes the mismatch between the visual, vestibular, and proprioceptive inputs (Brooks et al., 2010).

The present pilot study aimed to examine the feasibility, acceptance, and performance of a new, simple VR visual search task presented using an HMD, in right-hemispheric stroke patients and healthy controls. The first hypothesis was that the new VR cancellation task and the used device setup have high usability and satisfaction and that they do not lead to side effects. The second hypothesis was that the VR cancellation task can detect neglect.

## Materials and Methods

### Participant Recruitment and Demographics

**Figure 1:**
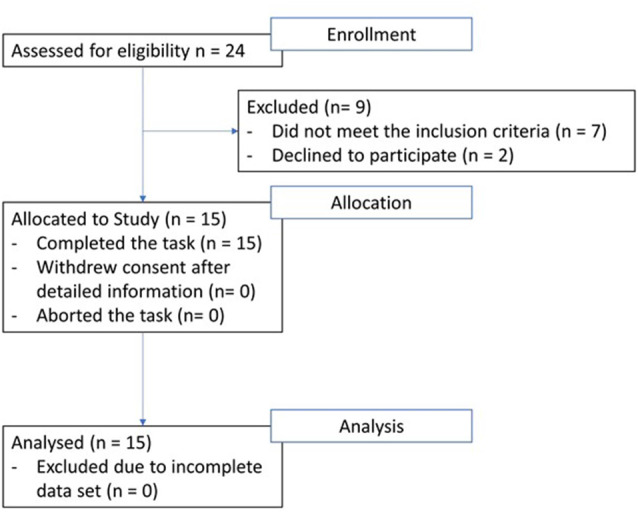
Patient flow-chart from enrolment to analysis.

Fifteen patients [mean age 67.1 years (range 50–84)] with right-hemispheric stroke were recruited from the neurorehabilitation units of the Kantonsspital Luzern and of the University Hospital in Bern in Switzerland. The inclusion criteria for the patient group were having had a first-ever stroke within the past 6 months and no other neurological or psychiatric disease (Figure 1).

The subjects of the stroke group were subdivided into a Neglect and a No-Neglect group according to their result in the paper-pencil cancellation task Sensitive Neglect Test (SNT). The 35 healthy controls were age-matched [mean age 69.0 years (range 54–84), *t*_(48)_ = 0.703, *p* = 0.486; Table 1].

**Table 1:** Overview of demographics in the groups.

	Stroke patients	Healthy controls
Group size	15	35
Unilateral spatial neglect (SNT)	10	0
Age (mean ± SD)	67.1 ± 10.5	69.0 ± 7.47
Gender (m/f)	7/8	28/7
Computer-Experience (1–100)	38 ± 32.0	46.4 ± 22.6
VR-Experience (yes/no)	0/15	1/34
Stroke type (Haemorrhagic/Ischemic)	3/12	—
Days since stroke	76.5 ± 36.0	—

*SNT, single neglect test; SD, standard deviation; VR, virtual reality*.

All participants answered a questionnaire about their computer experience and VR knowledge. No group-difference was found regarding computer experience (*t*_(48)_ = 1.06, *p* = 0.295) nor previous VR knowledge (*t*_(48)_ = 0.651, *p* = 0.518).

The instructions and aims of the study were explained in detail in advance and their understanding was confirmed by signing the informed consent forms. There was no monetary or non-monetary compensation for participation. Patients and healthy controls were recruited between 14th April 2018 and 11th September 2019. This study was approved by the Ethics Committee of the Canton of Bern, Switzerland, and has been conducted following good clinical practice guidelines and with the latest version of the Declaration of Helsinki.

### Apparatus and VR-Task

**Figure 2:**
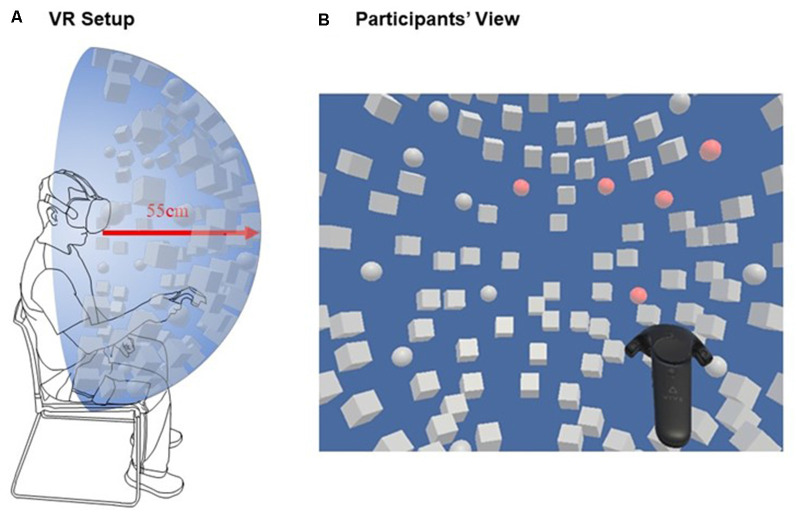
**(A)** Scheme of the subject wearing the head-mounted displays (HMD) and holding a virtual reality (VR) controller in the dominant hand overlaid with the targets and distractors. **(B)** Participant’s view, where not all objects were in the initial field of view but could be found by turning the head.

The virtual cancellation task, displayed using the HMD, showed a blue background and 120 objects (Figure 2), arranged within a hemisphere with a radius of 0.55 m. The horizontal range of the hemisphere had 160° visual angle, the vertical range of 120° visual angle. Before starting the task, the hemisphere center was aligned with the head’s and the trunk’s midsagittal plane of the participants. In the task, participants could move the head freely, but the trunk was stabilized by a chair.

The presented objects were white-colored spheres (20) and cubes (100), which changed their color to red when touched with the controller (i.e., when “canceled”). The target spheres were placed symmetrically with respect to a vertical midline. The distractor cubes were dispersed randomly between the target spheres, and therefore asymmetrically distributed with respect to the vertical midline.

The goal of the task was to find all spheres (targets), by touching them as fast as possible with the hand-held controller. At the same time, it had to be avoided to touch the cubes (distractors). As feedback, any object turned red once it had been touched. Touching the same object several times did not affect (i.e., the object stayed red as per the first time it was touched). The task ended after the participant told the experimenter that he/she had found all target objects. No other instructions were given during the task (i.e., the participants were not told how many targets had been identified correctly).

To calculate the CoC from the in-task performance, because of the spherical 3D configuration, the horizontal angles between the midline and the targets were used, i.e., not their cartesian coordinates. To quantify the in-task performance regarding the speed of task solving, the mean amount of targets canceled per minute was calculated.

The VR setup consisted of an HMD and a hand-held controller (Figure 2). A mobile Gaming-Laptop (HP-Omen, graphic card NVIDIA GTX1050 and CPU Intel i7) was used to run the software programmed with Unity3D (Haas, 2014). The resolution of the HMD (HTC Vive, High Tech Computer Corporation, Taoyuan, Taiwan) was 2,160 × 1,200 pixels (Full HD), with a horizontal field of view of 110° and a frame rate of 90 Hz.

### Procedure

After explaining the purpose of the study, the task was described in detail to the participants; moreover, they were presented with an exemplary image of the view through the HMD, in which the targets and distractors were shown. The participants were seated, were wearing the HMD that was fitted to their head, and were holding a VR controller in the right hand (Figure 2). The experimenter started the software and the participants could begin to search for targets as soon as the objects appeared all at once. Once the participant declared to have found all targets, the experimenter stopped the software. After having finished the task, all participants were asked to fill out a questionnaire on usability, negative effects, and general demographic data. The questionnaire was slightly adapted, for our study, from Gerber et al. (2019a). The content regarding usability was based on the System Usability Scale (SUS; Brooke, 1996), the content regarding side effects on the Simulator Sickness Questionnaire (SSQ; Kennedy et al., 1993). The questions from the SSQ were divided into three categories: Nausea (general discomfort, stomach awareness, sweating), oculomotor problems (eye strain, headache), and disorientation (dizziness).

The paper-pencil cancellation task to assess the neglect severity was the commonly used SNT (Reinhart et al., 2016) consisting of two parts, a single and a dual part. As the dual version, in which the subject has to find two kinds of targets, is much more difficult and exhausting, only the single version (only one kind of target) was used. In the SNT—single version, a total of 40 targets have to be found among 240 distractors. The cut off for the allocation into the Neglect or No-Neglect subgroup was a CoC > 0.081 (Reinhart et al., 2016).

Both, SNT and the VR cancellation task were performed within 1 day.

### Statistical Analyses

If not mentioned otherwise the assumption of the equality of variances (for *t*-test or ANOVA) was tested with the Leven’s test. In all cases of the comparison of the demographics and the ratings, an unpaired two-sided *t*-test with equal variances was used. For *post hoc* analysis of the ANOVA Bonferroni corrected pairwise *t*-test was used. If the assumptions of equal variances were not met, the nonparametric Kruskal–Wallis rank-sum test was used. For *post hoc* comparisons Bonferroni corrected pairwise Wilcox -test was used.

No significant differences between the two stroke-subgroups were found regarding age, experience with VR, or experience with the computer (*t*-test: *p* > 0.05 for all mentioned variables). Correspondingly, the two stroke-sub-groups (Neglect and No-Neglect) were joined into one single group to analyze the results of the questionnaires. For further analysis, the two stroke-subgroups were not joined, and the data were compared between the three groups (Healthy, Neglect, No-Neglect). This further analysis included the comparison between the CoC in the VR cancellation task and the SNT, as well as two measures addressing the in task-performance: the number of targets per time and the number and distribution of targets within the first 5 s.

For the calculation of the correlation between the performance in the paper-pencil and the performance in the VR cancellation task, Pearson’s correlation was used.

The data were analyzed with Matlab19b (MathWorks Inc, 2019 and R (R Core Team, 2018). Further study data are available upon request to the corresponding author.

## Results

### Acceptance, Usability, and Side Effects

**Figure 3:**
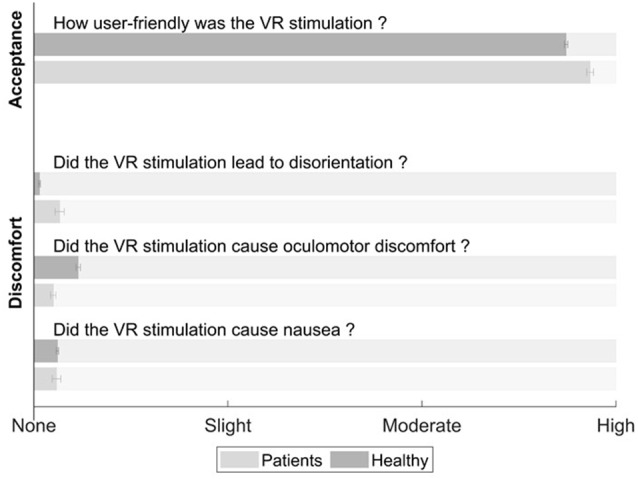
Overview and comparison of the questionnaire result from the System Usability Scale (SUS) and the Simulator Sickness Questionnaire (SSQ). The gray bars represent the mean values in the healthy controls, light gray represents the mean values for the stroke patients. No significant differences were found between the groups. The whiskers represent the mean standard error.

For the analysis of the results of the questionnaires, the data of both stroke patient groups were analyzed as one group. The findings (Figure 3) on usability (SUS) and side effects (SSQ; range 0–3) showed no significant differences between the two groups (healthy controls, stroke patients) regarding acceptance, usability or adverse effects. The usability itself was rated high, i.e., close to the maximum score, in both groups (healthy controls 2.743 ± 0.300, stroke: 2.867 ± 0.265; *t*_(48)_ = −1.38, *p* = 0.173).

Symptoms of discomfort such as nausea (healthy controls: 0.121 ± 0.205, stroke patients: 0.117 ± 0.339; *t*_(48)_ = 0.061, *p* = 0.951), oculomotor problems (healthy controls: 0.229 ± 0.408, stroke patients: 0.100 ± 0.207; *t*_(48)_ = 1.15, *p* = 0.255) or disorientation (healthy controls: 0.029 ± 0.169, stroke patients: 0.133 ± 0.352; *t*_(48)_ = −1431, *p* = 0.159) were rated close to the minimum score in both groups. In absolute numbers, only three healthy controls and one stroke patient reported moderate side effects.

### Center of Cancellation

**Figure 4:**
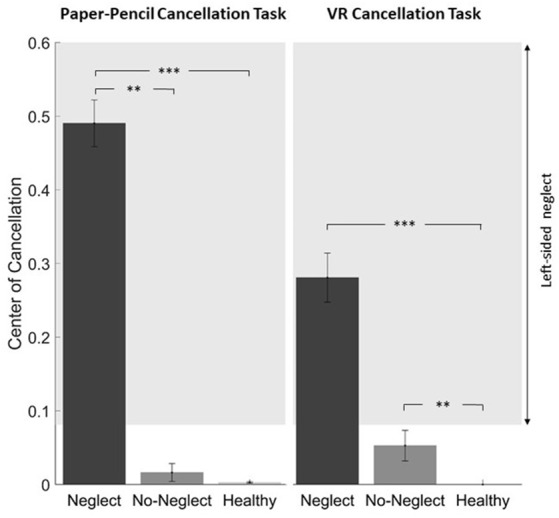
Center of Cancellation in the three groups (Neglect: dark-gray, No-Neglect: gray, Healthy: light-gray) in the paper-pencil (left side) and the VR cancellation task (right side). The asterisks represent the level of significance: **p < 0.01, ***p < 0.001. The whiskers represent the mean standard error. The Center of Cancellation represents the center of mass of the spatial distribution of detected targets and is standardized between plus and minus one (Rorden and Karnath, 2010).

Figure 4 shows the CoC for the three groups (Neglect, No-Neglect, Healthy) in the two performed tasks: on the left side, the CoC calculated from the performance in the paper-pencil cancellation task, and, on the right side, the one calculated from the performance in the VR cancellation task.

The Kruskal–Wallis rank-sum test showed a significant difference between the groups in both tasks (SNT: $\chi_{\left(2\right)}^{2}$ = 24. *p* < 0.001, VR: $\chi_{\left(2\right)}^{2}$ = 14. *p* = 0.001). *Post hoc* tests (Figure 4) revealed that the Neglect group showed a significant shift of the spatial distribution of the detected items towards the right, as assessed using the CoC (Rorden and Karnath, 2010). The *post hoc* pairwise Wilcox-test in the VR cancellation task showed a significant difference between the Neglect group and the healthy controls, as well as a significant difference between the No-Neglect patients and the healthy controls.

**Figure 5:**
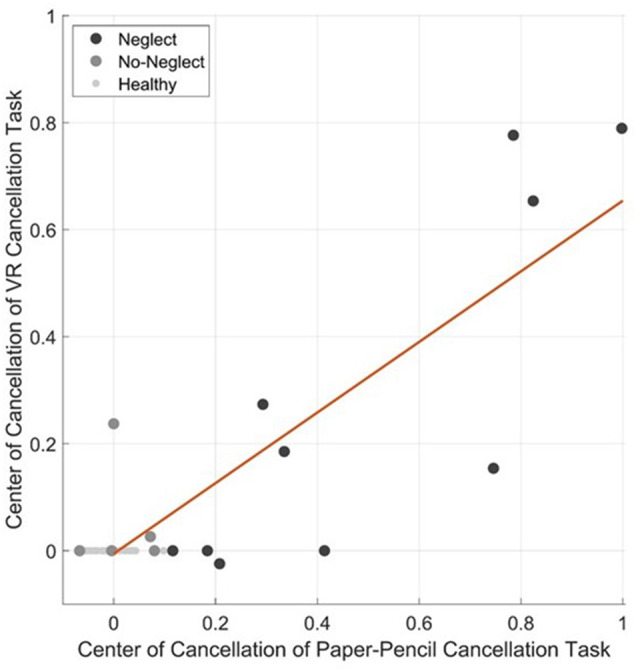
Correlation plot with regression line (in red) of the Center of Cancellation calculated in the VR cancellation task and the paper-pencil cancellation task for all participants.

An overview of the distribution of the CoC data and their relationship calculated from the two tasks is shown in Figure 5. The correlation between the two measures was *r* = 0.821 with *t*_(48)_ = 11.95, *p* < 0.001.

### Task Performance

**Figure 6:**
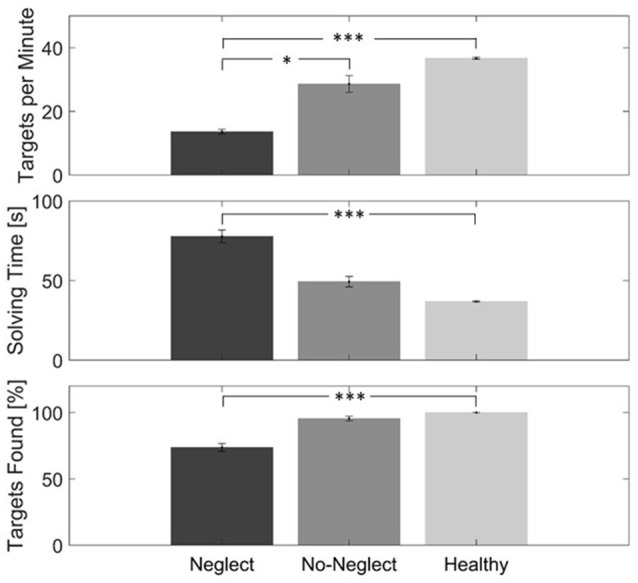
In-task performance data of the VR cancellation task of the three groups (Neglect: dark-gray, No-Neglect: gray, Healthy: light-gray). The top graph shows the number of targets per minute. The middle graph displays the mean solving time each group had. The bottom plot shows the percentage of found targets in each group. The asterisks represent the level of significance: *p < 0.05, ***p < 0.001. The whiskers represent the standard error.

The two raw measures, total time and percent targets found, were significantly different between the three groups (total time: $\chi_{\left(2\right)}^{2}$ = 13.5. *p* = 0.001/percent targets found: $\chi_{\left(2\right)}^{2}$ = 27.4. *p* < 0.001). For both measures, *post hoc* tests showed a significant difference between the healthy controls and the Neglect group (Figure 6). When these two measures were combined, i.e., considered as the number of targets identified per minute, there was as well a significant difference between the three groups ($\chi_{\left(2\right)}^{2}$ = 21. *p* < 0.001). In this case, the *post hoc* tests showed a significant difference between the Neglect group and the two other groups, but not between the control group and the No-Neglect group (Figure 6).

**Figure 7:**
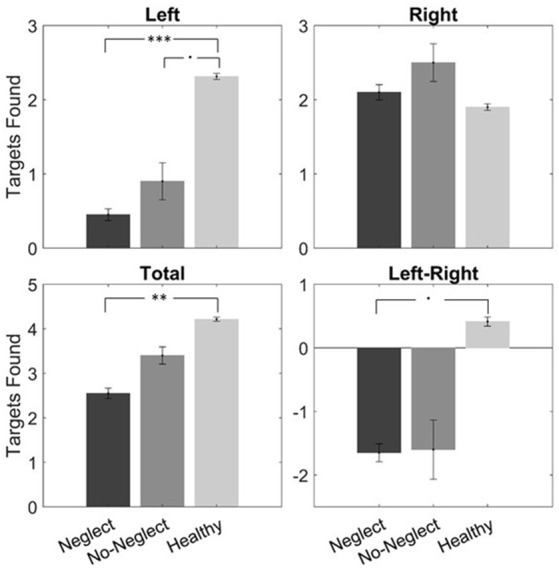
In-task performance data of the VR cancellation task of the three groups (Neglect: dark-gray, No-Neglect: gray, Healthy: light-gray) of the first 5 s. In the top row, the number of targets found on the left and the right side is shown. In the bottom row, the total number of targets and the left vs. right difference is presented. The whiskers represent the standard error. The asterisks represent the level of significance after Bonferroni correction: ^•^*p* < 0.1, **p < 0.01, ***p < 0.001.

Figure 7 shows the performance for the total number of targets canceled within the 5 first seconds on the whole array, on the left side, and the right side as well as the difference between the number of targets canceled on the left side minus the targets on the right side. Table 2 shows the results of the ANOVA for each of the four target positions between the three groups. There was a significant difference between the three groups concerning the total number of canceled targets, the number of targets canceled on the left side, and the right-left difference but not for the number of targets canceled on the right side. The *post hoc* tests (Figure 7) showed a significant difference between the healthy controls and the Neglect group for the total number of targets and the targets on the left side. There was as well a trend towards a difference in the number of targets canceled on the left side between the healthy controls and the No-Neglect group.

**Table 2:** Distribution of the number of targets found in the first 5 s.

	Neglect	No-Neglect	Healthy controls	ANOVA
Left	0.450 ± 0.798	0.90 ± 1.24	2.31 ± 1.46	*F*_(2,47)_ = 8.78***
Right	2.10 ± 1.02	2.50 ± 1.27	1.90 ± 1.54	*F*_(2,47)_ = 0.42, n.s.
Total	2.55 ± 1.65	3.40 ± 0.96	4.21 ± 1.58	*F*_(2,47)_ = 5.22**
Left-Right	−1.65 ± 1.41	−1.6 ± 2.33	0.414 ± 2.55	*F*_(2,47)_ = 3.97*

The values in the three groups represent the mean and its standard deviation. n.s. = p < 0.05, *p < 0.05, **p < 0.01, ***p < 0.001.

## Discussion

### Main Results

In line with the first hypothesis, the results of the questionnaires revealed a very high acceptance of the VR system and the VR cancellation task. Additionally, we could prove that the VR system was easy to use and that neither healthy controls nor stroke patients experienced any relevant discomfort using it.

For both tests, the paper-pencil and the VR cancellation tasks, a significant difference between groups was found. Neglect patients showed worse performance than No-Neglect patients and healthy controls. No significant difference was found between No-Neglect patients and controls.

### Acceptance, Usability and Side Effects

Firstly, the ratings of acceptance and usability were very high and close to the maximum score. Gerber et al. (2019a) found similar values, using a comparable technical setup with critically ill patients in the intensive care unit (ICU). Secondly, the ratings in the SSQ showed very low values, thus reflecting a safe device regarding cybersickness. These values are, again, in a similar range as the ones reported by Gerber et al. (2019a,b) in two studies. Some of the moderate side effects reported by three healthy controls (namely sweating), were most probably closely related to the measurement location and situation, as the measurements took place during winter in a very well heated room.

Earlier studies using HMD and VR for the assessment of neglect had also found the high acceptance of the setup and its feasibility (Gupta et al., 2000; Kim et al., 2004; Yasuda et al., 2017; Ronchi et al., 2018). However, the present study has several advantages. For example, a new full immersive VR technology was used with an HMD. Correspondingly, the relevant content was built around the participants, encouraging them to move their head to explore the full frontal view field, since all presented stimuli were relevant to solve the task. Furthermore, high usability was achieved, although none of the participants had neither a prior knowledge in VR nor a very high computer experience. Even though our pilot study has a similar sample size with respect to the study by Yasuda et al. (2017), our study additionally included healthy controls and stroke patients without neglect as a control group.

### Center of Cancellation

Regarding the CoC, two main findings shall be discussed.

First, the neglect symptoms in the Neglect group were more severe when measured by the paper-pencil cancellation task than when measured by the VR cancellation task. The inter-test correlation between different cancellation tasks typically lays between 0.4 and 0.8 (Halligan et al., 1989); correspondingly, a difference was to be expected. The correlation of *r* ≈ 0.8 between the CoC from the VR cancellation task and the SNT lies within the expected range and therefore the VR cancellation task seems to detect neglect as reliably as the used paper-pencil cancellation task. Another relevant point that may contribute to the difference is, of course, the different configuration (2D vs. 3D), as well as the different number of targets and distractors and their size. Similar differences in difficulty occur between the SNT single and dual, where subjects have more problems solving the SNT dual-task with twice the number of targets compared to the SNT single (Reinhart et al., 2016). More distractors and targets typically worsen the neglect symptoms, reflected in a Center of Cancellation further away from zero (Sarri et al., 2009; Cazzoli et al., 2012).

Second, the results have to be interpreted with precaution due to the low sample size. No significant difference could be found between the Neglect and the No-Neglect group within the VR cancellation task. From the overview in Figure 5, it can be assumed that the significant difference between the healthy controls and the No-Neglect group is the result of a single outlier. This, together with the fact that four of the subjects from the Neglect group found all targets in the VR cancellation task, is representative of the heterogeneity of the stroke population.

With four “missed” neglect patients according to the VR CoC alone, the current version of the VR cancellation shows a lower sensitivity than the paper-pencil SNT cancellation task. Keeping in mind that the primary aim of our preliminary study was to assess the usability acceptance of the VR setting in stroke patients in the following we will discuss some potential factors that may have led to this difference. First, it has been shown that neglect patients’ performance in cancellation tests is highly dependent on the number of distractors and targets presented, i.e., the higher the number of distractors, the more severe the neglect signs, and the higher the sensitivity of the test (Eglin et al., 1994; Husain and Kennard, 1997). The current version of our VR task included a lower number of distractors and targets (100 and 20, respectively) than the SNT paper-pencil test (40 and 240, respectively). Given the very high acceptance of our VR setup, future studies should aim to reapply the same setup and to manipulate the identity and number of stimuli to maximize the sensitivity of the task. Other important factors may be the duration and the novelty of the task. Most patients solved the SNT paper-pencil test within 5 to 10 min, whereas all patients performed the VR cancellation task in less than 5 min. Since neglect patients are known to have difficulties in sustaining attention over time (Robertson et al., 1995) a longer testing duration may lead to more prominent neglect signs. Moreover, the VR setup and task represented a new experience for most patients; one can postulate that this novelty may increase motivation and alertness, both factors have been shown to lead to temporary amelioration of neglect signs (Soto et al., 2009; Russell et al., 2013; Chandrakumar et al., 2019).

### Task Performance

The two measures total time and percentage of found targets showed a significant difference between the healthy controls and the Neglect group, but no significant difference between the No-Neglect group and the other groups was found. As the total time depends on the number of found targets, the number of targets per minute was calculated. Due to the low sample size, this, of course, has as well be interpreted with precaution. The Neglect group needed significantly more time per target than the other two groups. This can most probably be explained best by non- spatial attention deficits of neglect patients (Van Vleet and DeGutis, 2013). Although it was not exactly measured, we saw this pattern as well in the paper-pencil cancellation task.

Although the healthy controls and the No-Neglect group did not differ in the VR cancellation task or the SNT, considering the performance data within the first 5 s revealed a trend towards significance, suggesting different early search strategies in the groups. The No-Neglect group, as well as the Neglect group mainly started their search on the right side, whereas healthy controls did not prefer one side. This behavior has been described as an early, rightward orientation bias (Pflugshaupt et al., 2004; Azouvi et al., 2006), a sensitive measure for residual neglect. This suggests that the combination of the VR CoC and the behavior within the first 5 s as signs of early reorientation could improve the sensibility of the setup.

### Limitations and Outlook

As this study had a pilot character, only a few patients with different levels of impairment were included. On the one hand, the wide variety of participants’ characteristics are desired for testing acceptance, usability, and side effects. On the other hand, this makes the interpretation of the task performance data more difficult. Additionally, as the SNT was performed up to 1 day before the VR task, two factors have to be considered. First, as we did not randomize the order, a training effect between the two measures cannot be excluded. Second, neglect severity underlies spontaneous fluctuations (Li and Malhotra, 2015). Therefore, future studies should consider a randomized test design with a larger sample size to control for such confounders.

For the characterization and the allocation of the stroke patients, only one paper-pencil cancellation task was used. Future studies should aim to recruit a larger group of neglect patients and assess the neglect symptoms using several methods also including, e.g., eye-tracking (Kaufmann et al., 2020) and the assessment of neglect in the activities of daily living with the Catherine-Bergego Scale (Bergego et al., 1995).

As the main focus of this study was on the usability and acceptance of the setup and the developed virtual environment, no eye-tracking was used, and the VR cancellation task was only implemented for testing neglect in near space. Correspondingly, our data do not allow us to comment on the relationship between unilateral spatial neglect behavior in near and far space. However, in future studies, a setup able to test neglect in both near and far space can be easily implemented in VR, especially because of its high usability and acceptance. Moreover, the implementation of eye-tracking for assessing visual exploration behavior during the task should be considered.

According to the literature, static cancellation tasks (even in VR) may not always be sensitive enough for detecting neglect (Dawson et al., 2008; Buxbaum et al., 2012). Therefore, future studies may also want to investigate the use of dynamic cancellation tasks in a 3D environment as a diagnostic tool for neglect. This dynamic component can easily be implemented in a VR setup, which is not the case in a paper-pencil setting.

## Conclusions

The results of this pilot study show that the setup and the newly developed VR cancellation task were easy to use, highly accepted, and did not provoke any relevant side effects in stroke patients with and without neglect. Overall, the questionnaire results and the high correlation between the CoC calculated from the performance in the VR cancellation task and the paper-pencil task support the suitability of the test setup and the task as a new tool for detecting neglect in stroke patients. Based on these findings and the fact that dynamic aspects and everyday scenarios can be easily implemented, tasks in VR can also potentially help in the rehabilitation of neglect in the future.

## Data Availability Statement

The datasets generated for this study are available on request to the corresponding author.

## Ethics Statement

The studies involving human participants were reviewed and approved by Kantonale Ethikkommission für die Forschung, Murtenstrasse 31, 3010 Bern. The patients/participants provided their written informed consent to participate in this study.

## Author Contributions

SK, DC, BK, RM, TNy, and TNe designed the study. SK and SG developed the tool and the setup. SK analyzed the data set. SK, BK, DC, and TNe wrote the manuscript. All authors approved the final manuscript.

## Conflict of Interest

The authors declare that the research was conducted in the absence of any commercial or financial relationships that could be construed as a potential conflict of interest.
